# Midterm functional performance following open surgical repair of acute Achilles tendon rupture

**DOI:** 10.1007/s00402-020-03746-3

**Published:** 2021-01-23

**Authors:** Markus Wenning, Marlene Mauch, Albrecht Heitner, Paul Streicher, Ramona Ritzmann, Jochen Paul

**Affiliations:** 1 Rennbahnklinik, Kriegackerstr. 100, CH-4132 Muttenz, Baselland Switzerland; 2 Department of Orthopedic and Trauma Surgery, University Medical Center Freiburg, Faculty of Medicine, Albert-Ludwigs University of Freiburg, Hugstetter Str. 55, 79106 Freiburg, Germany; 3 Department of Sport and Sport Science, University of Freiburg, Freiburg, Germany

**Keywords:** Achilles tendon rupture, Anatomical reconstruction, Functional performance testing

## Abstract

**Introduction:**

Various impairments such as soleus atrophy and consecutive functional deficits in end-range plantarflexion have been described in surgical repair of acute Achilles tendon rupture. The aim of this study was to assess the functional performance at midterm following open surgical repair.

**Materials and Methods:**

This cross-sectional study includes *n* = 52 patients which were tested on average 3.5 ± 1.4 years postoperatively using three different functional performance tests and patient-reported outcome measures. Two different surgical techniques (anatomical repair = AR vs. conventional repair = CR) were compared in a subanalysis. The testing included isokinetic strength testing, a novel setup of heel-rise testing using a marker-based 3D motion analysis system and a gait analysis.

**Results:**

At an average 3.5 years post-surgery, there is a persisting deficit in plantarflexion strength of 10.2%. Moreover, analysis of maximum peak torque angle and strength deficits according to the plantarflexion angle revealed that these deficits are not equally distributed across the range of motion. AR results in a significantly smaller deficit at 10° of plantarflexion compared to CR (13.9 vs. 29.9%, *p* < 0.05). This reflects into the functional performance during different modalities (static vs. dynamic) in this novel method of heel-rise testing.

**Conclusion:**

In summary, there are persisting functional deficits at > 3 years following Achilles tendon repair which range from strength deficits to specific impairments of functional performance e.g. during heel rise. Anatomical reconstruction is associated with an improved functional performance potentially due to a more symmetric strength during end-range plantarflexion which transfers into a higher satisfaction during athletic activities.

**Level of evidence:**

III, retrospective cohort study

**Supplementary Information:**

The online version contains supplementary material available at 10.1007/s00402-020-03746-3.

## Introduction

With the Achilles tendon (AT) ruptures showing a steady increase in incidence over the past decades [1], there is ongoing debate on the optimal treatment algorithm [2–4]. Few recent randomized controlled studies have compared surgical to nonoperative treatment [4–7] and also conservative to accelerated rehabilitation [8, 9]. Summarizing the latest evidence, there is a strong tendency toward surgical treatment with accelerated rehabilitation in the young athlete, due to a quicker return to sports, less muscle atrophy, and improved functional performance coupled with a lower risk of re-rupture [2, 5, 10].

Regarding lower leg performance, it was recently shown that soleus atrophy is more common in non-surgical treatment resulting in greater strength deficits, especially in end-range plantarflexion [5]. However, also in surgical treatment there is a relevant postoperative deficit in plantarflexion strength ranging from 12 to 30% [11, 12]. In a clinical setting, this end-range deficit is often evaluated by heel-rise height and heel-rise work, which has been shown to correlate with patient-reported outcome (PRO) and physical performance [13–15]. Especially in an athletic population, it is necessary to maintain the end-range plantarflexion strength in order to allow for the full range of performance as for example during sprinting and jumping [16, 17].

When deciding to perform surgical repair after acute AT rupture, there are several surgical techniques: Open repair using variations of end-to-end Kessler suture, augmented repairs, different techniques of mini-open or percutaneous repair, and anatomical reconstruction have been described [18–21]. The differences, especially in early and midterm functional performance, have not shown significant differences between these techniques, and there is only a paucity of studies comparing different techniques [6, 12, 18]. Only the rate of major complications like necrosis has been shown to be less in percutaneous repair, while re-rupture rates and sural nerve irritation may be increased [18]. Apart from traditional Kessler’s suture-type repairs which is a mere adaptation of the ruptured ends, a reconstructive technique of the ruptured AT was first established by Segesser et al. [20]. Going beyond the traditional Kessler’s suture-type repair, this has been stated to be respecting the original, twisted anatomy, resisting the asymmetric loading of the tendon, and it dedicates special attention to the integration and pretensioning of the soleus muscle [20, 22].

In terms of patients’ satisfaction, AT rupture is associated with a highly acceptable outcome averaging midterm Achilles Tendon Rupture Scores (ATRS) above 85/100 [23, 24]. Neither rehabilitation scheme (accelerated vs. traditional) nor surgical vs. non-surgical treatment seem to have a significant advantage in the average patient [23, 24].

The aim of the present study was to evaluate midterm functional performance after AT repair. Derived from the available literature, our main hypothesis was that there will still be end-range isokinetic plantarflexion deficits which will further reflect into the dynamic performance testing like heel rise and gait analysis [5, 25, 26].

The secondary aim was to assess whether the repair of the AT as in an “anatomical reconstruction” technique resulted in superior objective outcomes compared to the traditional repair technique. From previous studies, we hypothesized that the explicit integration of the soleus muscle and maximum pretensioning will reduce the end-range deficit in plantarflexion strength compared to conventional Kessler’s suture [22]. In an explorative approach, we aimed to assess whether this would lead to improved subjective function using PRO measures.

Note: In this article, we focused on the functional measures and strength performance, while the differences in the neuromechanical activation pattern will be reported in a separate publication.

## Methods

The study was carried out in accordance with the declaration of Helsinki, and it was approved by the local ethics committee EKNZ 2017-02206. All patients declared written informed consent prior to inclusion into the study.

This is the first part of results from a large and multivariate cross-sectional comparative study. We assessed the midterm functional performance of *n* = 52 patients that had undergone surgery for acute traumatic AT rupture in a biomechanical testing setup. Inclusion criteria, as deducted from the literature [1, 2], were acute AT open repair in two centers involved in the study, fewer than ten days after rupture, male gender, and age < 60 years. Exclusion criteria were other injuries to the lower extremities such as tendon ruptures, fractures requiring surgical repair or injury to the contralateral tendon, re-rupture, a history of neurological impairments such as disk herniation and/or sciatica and diagnosed diabetes mellitus. All tests were performed in a single session and supervised by two examiners experienced in biomechanical testing. The neuromechanical activation pattern (EMG activation), which was recorded simultaneously, will be reported and discussed separately.

### Patients

**Fig. 1:**
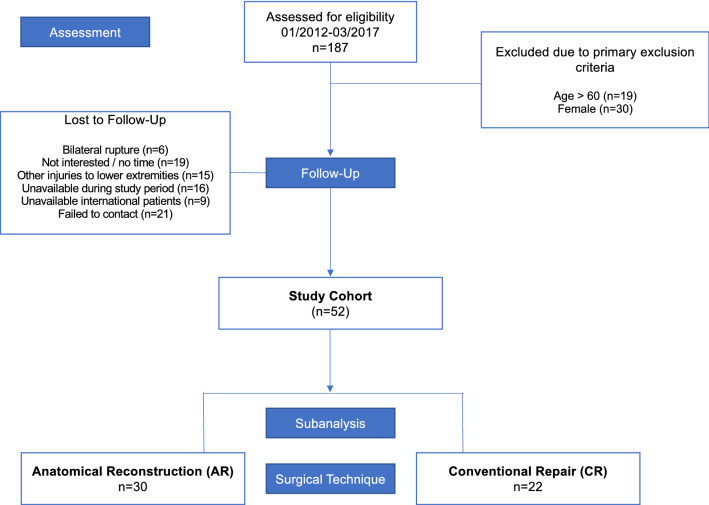
CONSORT flow-chart of patient recruiting

Recruitments were carried out at two centers in which all patients (*n* = 187) operated between January 2012 and March 2017 were assessed for eligibility, while the functional performance measurements were performed at the same biomechanical laboratory. Table 1 displays the characteristics of the study group as well as the subgroups, while Fig. 1 shows the flowchart of the recruiting process. The mean time between surgery and the study assessment was 3.5 ± 1.4 years. Of the potential 138 patients, 52 were willing to participate in the study; the details are listed in the flowchart.

**Table 1:** Baseline characteristics of the study group and subgroups

	Total study group (*n* = 52, Mean ± SD)	Subgroups		Sign (AR vs. CR)
		AR (*n* = 30)	CR (*n* = 22)	
Follow-up (y)	3.45 ± 1.4	3.7 ± 1.5	3.1 ± 1.4	n.s
Height (m)	1.81 ± 0.6	1.81 ± .06	1.81 ± 0.07	n.s
Weight (kg)	88.7 ± 11.7	88.7 ± 9.7	88.8 ± 14.7	n.s
Age at surgery (y)	41.0 ± 9.8	40.5 ± 10.9	41.8 ± 8.4	n.s
Right leg injured	44.2%	46.7%	40.9%	n.s
Dominant leg injured	52.0%	53.3%	50.0%	n.s

*AR* anatomical reconstruction, *CR* conventional repair, *n.s.* not significant

AT rupture was diagnosed using MRI imaging or sonography. All patients received physical therapy and followed a standardized rehabilitation protocol. All patients had finished their rehabilitation before enrollment in the study and had gone back to sportive activities including running and jumping.

### Surgical technique of anatomical reconstruction

**Fig. 2:**
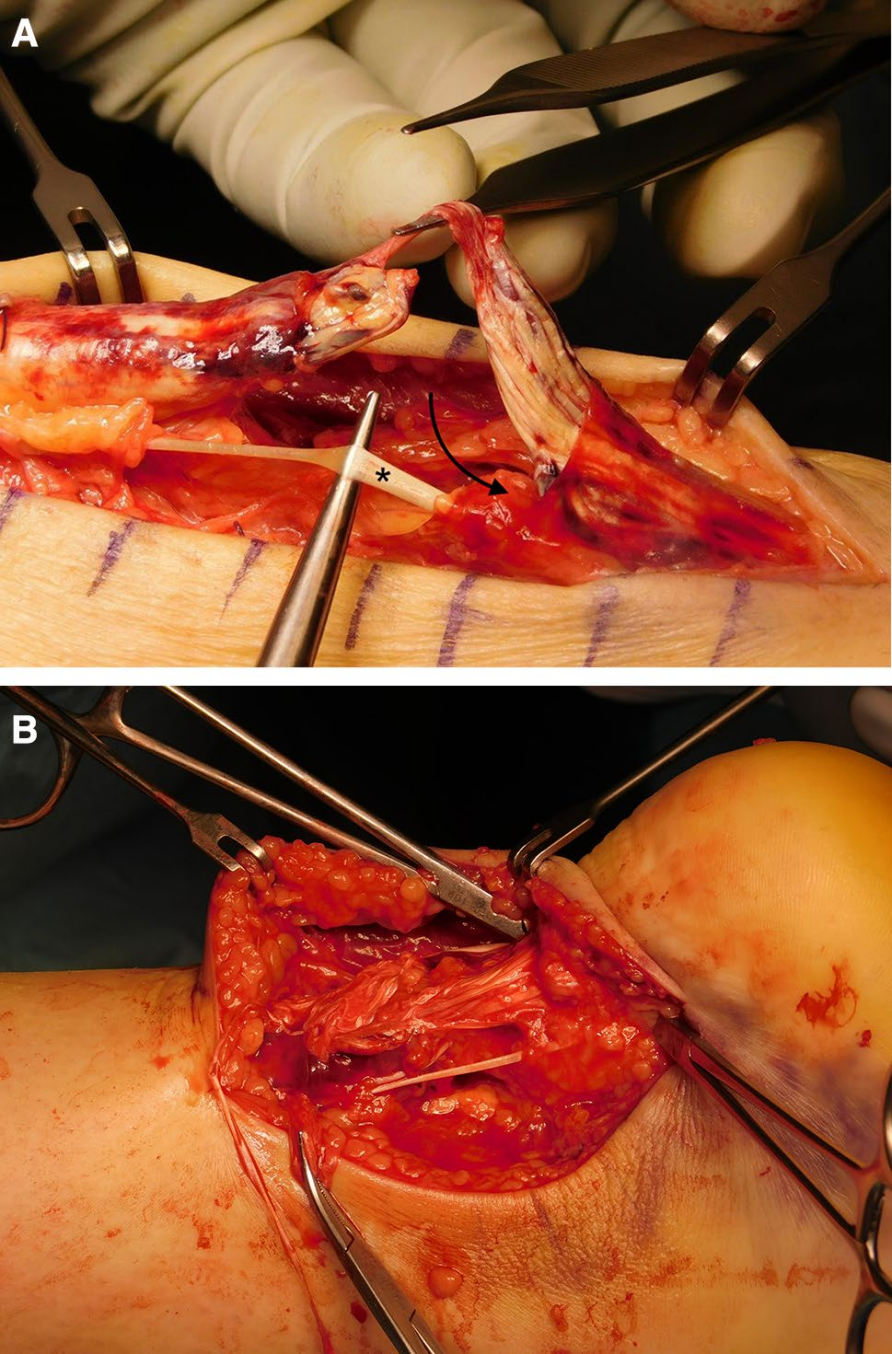
**a** Intraoperative display of the rupture site on a left foot, where the soleus muscle is retracted proximally below the rupture (arrow) and the plantaris tendon which has been preserved during preparation (asterisk). **b** Intraoperative image of a right foot displaying the “twisting”-technique using the sharpened surgical clamps in place

The anatomical reconstruction of the AT is performed in prone position via a medial, para-achillary incision. After incision of the peritendineum, the rupture is identified. Care is taken to identify the M. plantaris tendon which is dissected by a closed tendon stripping device. The insertion at the calcaneus is left in place. Special attention is paid to the distal part of M. soleus which is often retracted in acute AT ruptures (Fig. 2a) Maximal tensioning of this muscle is important for the function of the M. triceps surae unit. Therefore, it is mobilized and sutured to its anatomical insertion at the most medial fibers of the calcaneal rupture end using resorbable sutures. Following the “pretensioning” of one part of the AT, the Mm. gastrocnemii parts (medial and lateral) are anatomically reconstructed to the distal part using a “twisting” technique. The ruptured fibers are threaded through the opposing end, strictly following the original anatomic course using a sharpened clamp (Fig. 2b). Finally, the M. plantaris tendon is used as an additional strengthening component and the reconstructed tendon is framed with it.

Instantly after surgery, a long ventral hardcast is applied for up to 2 weeks. This cast is limiting knee flexion to 30° and maximum plantarflexion at the ankle joint, protecting the sutured tendon.

Postoperatively, progressive weight bearing is applied according to local hematoma, pain, and swelling. Generally, full weight bearing with the foot in plantarflexion in a protective AT boot (e.g., Vario-Stabil Achillessehnenstiefel, Orthotech, Stockdorf, CH) can be achieved about 3–5 days after surgery. Wedges are used in the boot for reduction in tendon tension which are gradually removed.

### Surgical technique of conventional repair

The conventional repair was performed in prone position and used a small medial, longitudinal para-achillary skin incision about two cm proximal and distal to the rupture site. Following the skin incision, the peritendineum was incised to display the rupture. The main ends of the proximal and distal rupture were sutured with a 1–0 PDS or a FiberWire (*n* = 2) non-absorbable suture (Arthrex, Naples, FL, USA) in a double-row Bunnell technique.

Postoperatively, the shaft was immobilized in a plaster cast for four weeks before gradually increasing weight bearing and starting functional rehabilitation six weeks after surgery.

### Patient-reported outcome

The PRO was assessed using the ATRS, which is an established and reliable measure for Achilles tendon function [27]. Tegner activity scale was used to reliably estimate the patients’ level of postoperative sports performance [28]. Furthermore, we used visual analogue scales (0–10) to estimate general satisfaction with the current function of the injured ankle in a standardized question: “How satisfied are you with the function of the operated leg in your everyday life?” The second question focused on sportive activity: “How satisfied are you with the function of the operated leg during sportive activity including running and jumping?”.

### Isokinetic maximal strength

Peak isokinetic strength was assessed using an isokinetic dynamometer (Humac Norm, CSMi, Stoughton, MA, USA) according to the literature [29]. Patients were placed in prone position in full knee extension. The foot, thigh, and shank were tightly fixed to the footplate using Velcro straps. The patients wore their sports shoes with flat heels. The plantarflexion/dorsiflexion axis (best guessed by the intermalleolar axis) was placed so that it coincided with the rotational axis of the lever arm. Three warm-up trials were performed for familiarization. For data assessment, we used a protocol of concentric–concentric contractions at 30°/s angular speed, in the full range of motion (ROM) due to its high test–retest reliability [29] and validity [23]. Two sets of five repetitions at maximum effort and verbal encouragement were executed starting with the unaffected side.

Outcome parameters were average measures of peak torque and maximum peak torque angle in plantarflexion and dorsiflexion [5], as well as averaged torque values in plantarflexion angle in steps of 10° [25]. To account for intraindividual differences, the limb symmetry index (LSI in %) for peak torque (affected limb/unaffected limb *100) for plantarflexors and dorsiflexors was calculated.

### Heel-rise test

**Fig. 3:**
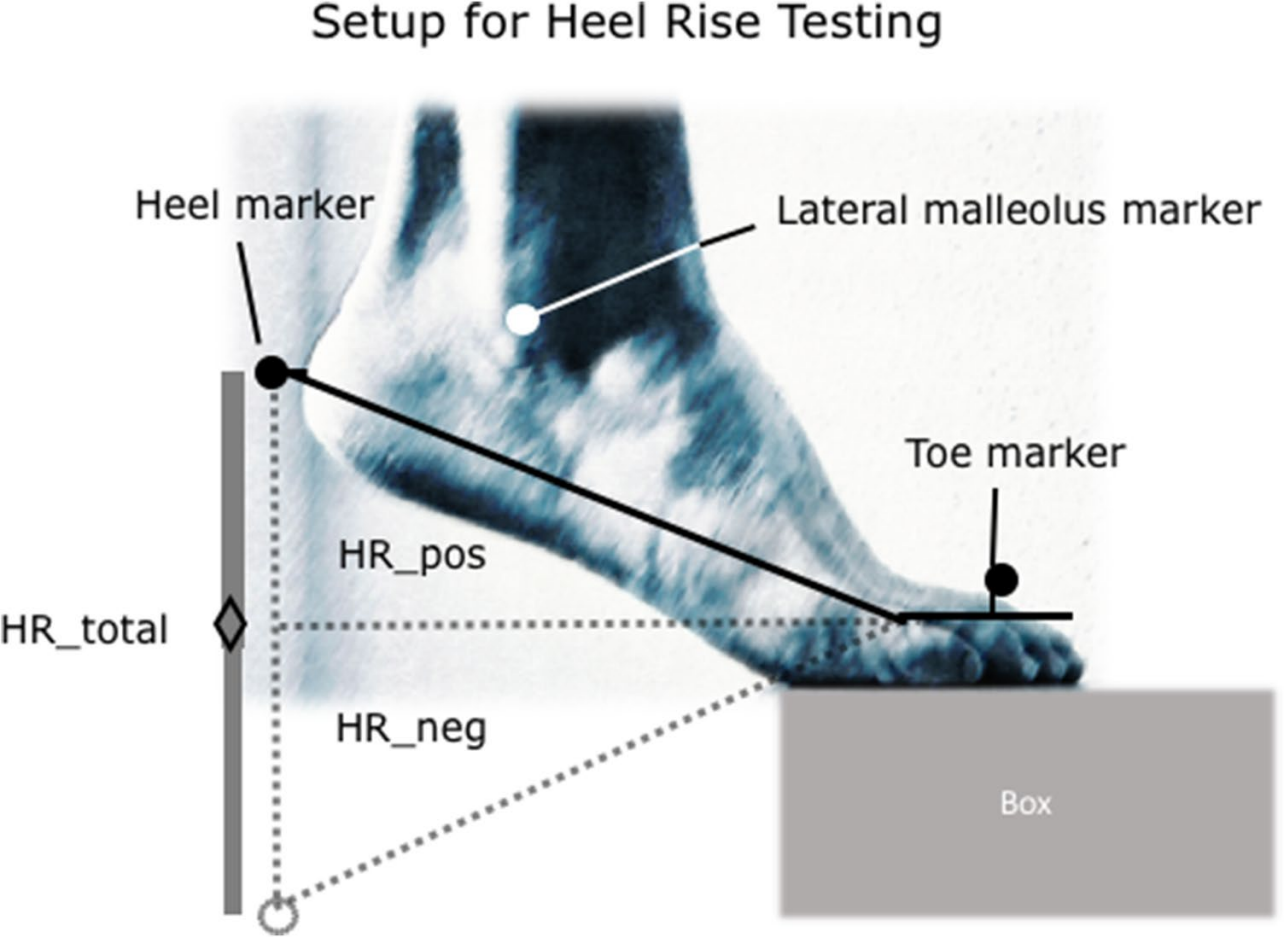
Setup for heel-rise testing using a three-marker-based analysis in 3D motion tracking

The setup for heel-rise test is displayed in Fig. 3. The patient was standing with the forefoot on a box of 30 cm height to allow for unrestricted dorsiflexion at the ankle joint. The patient was allowed to hold two fingertips against the wall for additional stabilization. They were instructed to go as high as possible on each heel rise before lowering down the heel to maximum dorsiflexion [15]. The range of motion of the ankle was measured using a three-dimensional motion analysis system with eight cameras (Vicon Motion Systems Ltd., Oxford, UK) at a frequency of 200 Hz, which has been shown to be of very high measurement accuracy and reliability. Three markers were placed on anatomical landmarks of the foot to analyze sagittal motion: (1) MTH II, (2) calcaneus, (3) lateral malleolus (see Fig. 3).

The protocol consisted of a *dynamic* testing including ten repetitions in bipedal stance followed by ten repetitions of dynamic one-legged heel rise always starting with the unaffected leg. Between bipedal and one-legged as well as before static testing, a resting period of 5 min was allowed. The *static* testing was performed holding maximum plantarflexion, e.g., heel-rise height for 30 s. The average heel-rise height between sec. 10 and 20 was used for analysis.

Post-processing of the data was performed using Nexus 2.7 (Oxford Metrics, Ltd., Oxford, UK). Three parameters were defined for analysis of functional strength performance: HR_total was the total height of the heel marker as a correlation of the maximum active dorsi-/plantarflexion. HR_pos was the maximum height achievable above the horizontal plane as compared to the marker on the forefoot, and HR_neg was defined as the minimum below this horizontal plane (s. Fig. 3).

### Gait analysis

Gait analysis was performed barefoot and wearing shoes while walking on a treadmill equipped with integrated pressure distribution platform (FDM-T, zebris Medical GmbH, Isny, Germany). Walking speed was adjusted to the individual’s preferred gait speed between 4 and 6 km/h as described in the current literature [30]. Based on the pressure data, maximum push-off force, the shift from posterior to anterior load in percent of the stance phase, and the duration of heel contact were calculated to assess the functional properties of the plantarflexors, particularly the AT, and to estimate gait symmetry. As previously described [17], this was done assuming that unequally distributed weight bearing leading to an increased time resting on the hindfoot could be attributed to avoidance of regular active weight bearing, propulsion, and plantarflexion strength during gait. Furthermore, these data were compared to the data acquired by an integrated force plate (Kistler AG, Winterthur, Switzerland) while walking in the 3D motion system for EMG analysis and triggering.

### Statistics

Before statistical comparison, outliers in limb symmetry indices were identified using box plot analysis and thereafter winsorized where necessary (< 0.5% of data). Descriptive analysis was performed for all variables, and significance was tested after checking for normal distribution using Shapiro–Wilk test. All variables were normally distributed except the visual analogue scales. To ease understanding of the results and subgroup analysis, we displayed the absolute values for the entire cohort and limb symmetry indices for AR vs. CR. The absolute values of the subgroups and the corresponding statistics are available in the online supplement. In general, with respect to the variety of testing modalities, a conservative statistical approach was chosen. For the primary outcomes, we performed a single-factor ANOVA to assess side-by-side differences. With respect to the nonlinearity of limb symmetry indices, we used a nonparametric Mann–Whitney *U* test to test for significance between the LSIs of surgical technique. Further, we performed an rm-ANOVA for the subanalysis of the strength measurements according to the plantarflexion angle with a Greenhouse–Geisser correction and Bonferroni-corrected post hoc analysis of group-wise comparisons. Spearman’s rho was calculated as a correlation measure to assess potential interaction.

In the supplementary data, we performed univariate two-factor ANOVA with the factors side (OP vs. non-OP) and group (AR vs. CR).

The level of significance was set at *p* < 0.05; however, to correct for multiple testing and resulting cumulative Type I error, Bonferroni–Holm corrections of the alpha level were applied where required (see results). Values are presented as means ± standard deviation.

The statistical analysis was performed using SPSS 24 (SPSS Inc., Chicago, USA). Graphical display was realized using Veusz v. 3.0.1 (Veusz Group, GNU-licensed, 2018).

## Results

### Patient-reported outcome

The PRO measures and sports level are displayed in Table 2.

**Table 2:** Patient-reported outcome measures of the entire group and the two subgroups

Parameter	Total group Mean ± SD	Subgroups		Sign
		AR	CR	
ATRS	87.0 ± 12.3	**90.1 ± 8.9**	**82.7 ± 14.8**	***p = 0.04***
Tegner scale	5.3 ± 1.7	5.4 ± 1.3	5.2 ± 1.7	n.s
VAS ADL	8.5 ± 1.4	**8.8 ± 1.3**	**8.1 ± 1.5**	***P < 0.05***
VAS sport	8.5 ± 1.9	9.2* ± 1.0	**7.6 ± 2.4**	***p < 0.05***

Bold indicates significant difference

*VAS* visual analogue scale (0–10), *AR* anatomical reconstruction, *CR* conventional repair, *ATRS* Achilles Tendon Rupture Score, *ADL* activities of daily living

Outcomes measured using ATRS (*p* = 0.04) and satisfaction during athletic activity (*p* < 0.05) reached statistically significant group differences with higher values for AR compared to CR. Group comparison for Tegner activity scale and patient satisfaction during activities of daily living remained insignificant.

### Isokinetic strength measurement

Isokinetic joint torques are displayed in Table 3, while Table 4 displays the limb symmetry indices according to the surgical procedure. The pairwise comparison in the rm-ANOVA between the different plantarflexion angles showed significant differences between all measurements except between torques at 10° of dorsiflexion. The maximum torque was also significantly different, and the maximum peak torque angle between legs during dorsiflexion was significantly greater on the operated side, equaling an MPTA at a more plantarflexed foot.

**Table 3:** Outcomes of isokinetic strength measurement

Parameter	OP	NOP	(rm-)ANOVA
MPTA PF (°)	− 8.1 ± 5.1	− 6.1 ± 3.7	*F*(1,101) = 3.51, *p* = 0.064
FMax PF total (Nm)	**140.7 ± 29.7**	**149.6 ± 33.3**	***F(1,100) = 5.78, p = 0.018***
FMax @ 20° PF (Nm)	**37.6 ± 16.2**	**45.6 ± 19.6**	***F(1,89) = 4.13, p = 0.041, Eta***^**2**^** = 0.047**
FMax @ 10° PF (Nm)	**70.5 ± 21.6**	**86.8 ± 26.5**	***F(1,100) = 15.64, p = 0.002, Eta***^**2**^** = 0.10**
FMax @ 0° PF (Nm)	**110.5 ± 25.9**	**129.5 ± 32.9**	***F(1,100) = 15.54, p = 0.004, Eta***^**2**^** = 0.09**
FMax @ 10° DF (Nm)	112.7 ± 46.7	118.3 ± 43.2	*F*(1,97) = 1.34, *p* = 0.55, *E*ta^2^ = 0.004
MPTA DF (°)	**13.2 ± 4.5**	**15.3 ± 3.8**	***F(1,100) = 3.98, p = 0.49***
FMax DF total (Nm)	47.2 ± 9.5	44.4 ± 9.8	*F*(1,100) = .83, *p* = .36

Bold = significant side-to-side differences in single-factor ANOVA except for *F*Max-subanalysis with rm-ANOVA analysis

*DF* dorsiflexion, *FMax* maximum torque, *MPTA* maximum peak torque angle (negative values = dorsiflexion), *Nm* Newton meter, *NOP* non-operated leg, *OP* operated leg, *PF* plantarflexion, *ROM* range of motion, *SD* standard deviation

**Table 4:** Subgroup analysis of LSI according to plantarflexion angle

Parameter	Subgroups LSI in %	
	AR	CR
FMax Plantarflexion total (Nm)	92.7 (18.6)	87.9 (15.4)
FMax @ 20° PF (Nm)	89.5 (33.8)	79.8 (42.4)
FMax @ 10° PF (Nm)	**86.1 (32.7)**	**70.1 (17.9)**
FMax @ 0° PF (Nm)	85.1 (23.2)	79.7 (16.1)
FMax @ 10° DE (Nm)	80.5 (31.3)	93.7 (37.0)
FMax Dorsiflexion total (Nm)	104.5 (19.2)	109.7 (22.7)

Bold indicates significant difference at *p* < 0.05, Mann–Whitney *U* test

*sAR*  anatomical reconstruction, *CR *conventional repair, *PF* plantarflexion, *DC* dorsiextension, *Nm* Newton meter, *FMax* maximum torque, *LSI* limb symmetry index

**Fig. 4:**
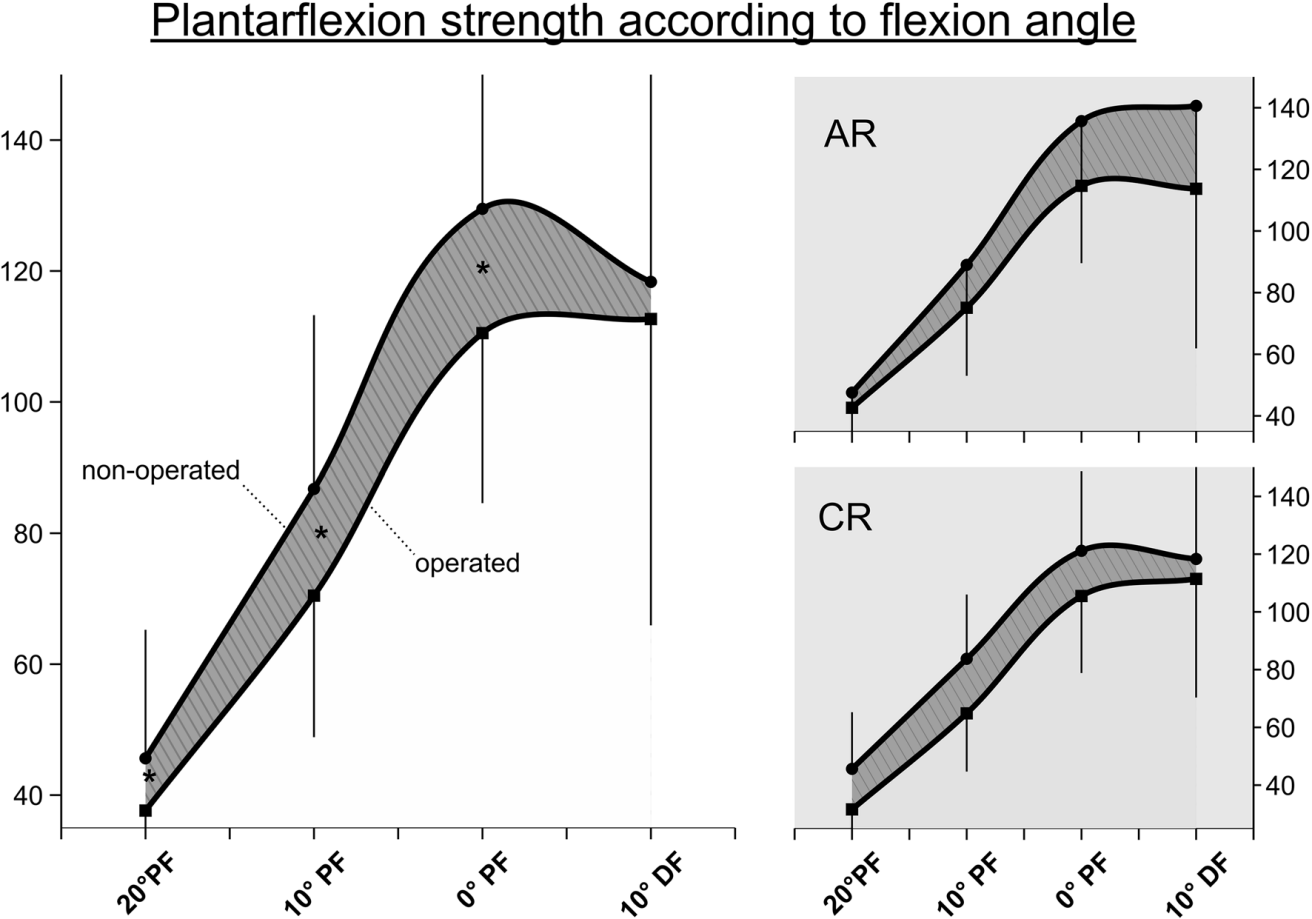
Limb symmetry index (%) for isokinetic strength measurement according to plantarflexion angle and surgical technique. *AR* anatomic reconstruction technique, *CR* conventional technique. *PF* Plantarflexion, *DF* Dorsiflexion

Analysis of the two subgroups according to the plantarflexion angle and the distribution of the strength deficits did show a diverging pattern (s. Fig. 4) with the difference in LSI reaching significance (*p* < 0.03, Mann–Whitney *U* test) at 10° plantarflexion.

### Heel-rise testing

**Fig. 5:**
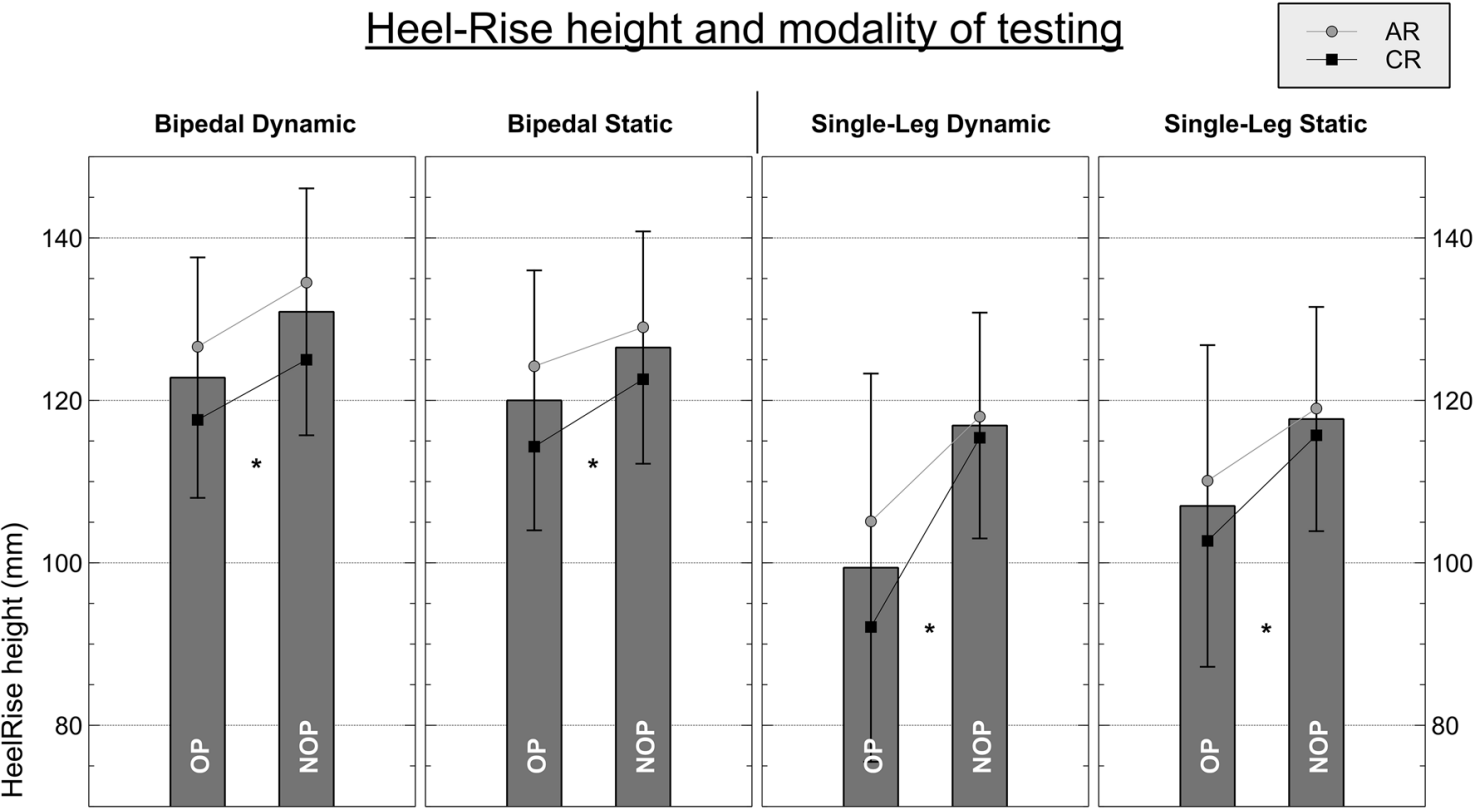
Graphical display of heel-rise testing using 3D motion analysis across the entire cohort (bars) and the two subgroups (lines). *AR* anatomic reconstruction technique, *CR* conventional technique. * = significant difference *p* < 0.025, *OP* operated leg, *NOP* non-operated leg

Figure 5 shows the results of the heel-rise testing, while Table 5 displays the LSIs across the subgroups. The absolute values of the total cohort and the subgroups can be found in the supplementary material.

**Table 5:** Results of the subgroup analysis in LSI during heel-rise testing according to the testing modality

Parameter	LSI in %	
	AR	CR
Bipedal static		
HR_tot	97.4 ± 6.1	96. + 94.8)
HR_pos	**96.7 (5.7)**	**91.0 (7.3)**
Bipedal dynamic		
HR_tot	97.6 (4.9)	97.0 (6.3)
HR_pos	**94.9 (6.3)**	**92.0 (7.5)**
HR_neg	104.6 (16.6)	106.9 (18.4)
Single-leg static		
HR_tot	93.7 (12.4)	91.1 (11.5)
HR_pos	**92.5 (15.2)**	**87.5 (16.5)**
Single-leg dynamic		
HR_tot	92.4 (11.4)	86.7 (11.8)
HR_pos	87.5 (14.7)	79.2 (17.2)
HR_neg	113.4 (37.1)	102.7 (35.1)

Bold indicates significant difference

We observed significant side-to-side differences across all four modalities when regarding the height achieved above toe marker (HR_pos, *p* < 0.017) and the total distance of travel (HR_tot,. *p* < 0.017). Contrarily, the negative turning point in dynamic testing showed no significant difference between legs or groups.

Additionally, there were significant differences between the subgroups of AR and CR where the observed side-to-side difference was significantly greater in CR compared to AR in both conditions (LSI *static* 91 vs. 96.7% and *dynamic* 92 vs. 94.5%).The limb symmetry index across the two subgroups during single-legged testing was only significantly different during static performance (AR 92.5% vs. CR 87.5%, *p* < 0.05).

### Gait analysis

During the gait analysis, no significant differences were found neither between the groups nor between the legs. When assessing barefoot vs. walking in shoes, we found no significant difference in the performance parameters except for a longer ground time of the hindfoot on the operated side (60.4 vs. 57.0% of the gait cycle). There were significant Spearman’s rho correlations between ground reaction forces and plantarflexion strength according to the plantarflexion angle with moderate correlations at 10° and 20° of plantarflexion and the push-off-force during barefoot and in-shoe gait analysis.

### Correlation analyses

**Fig. 6:**
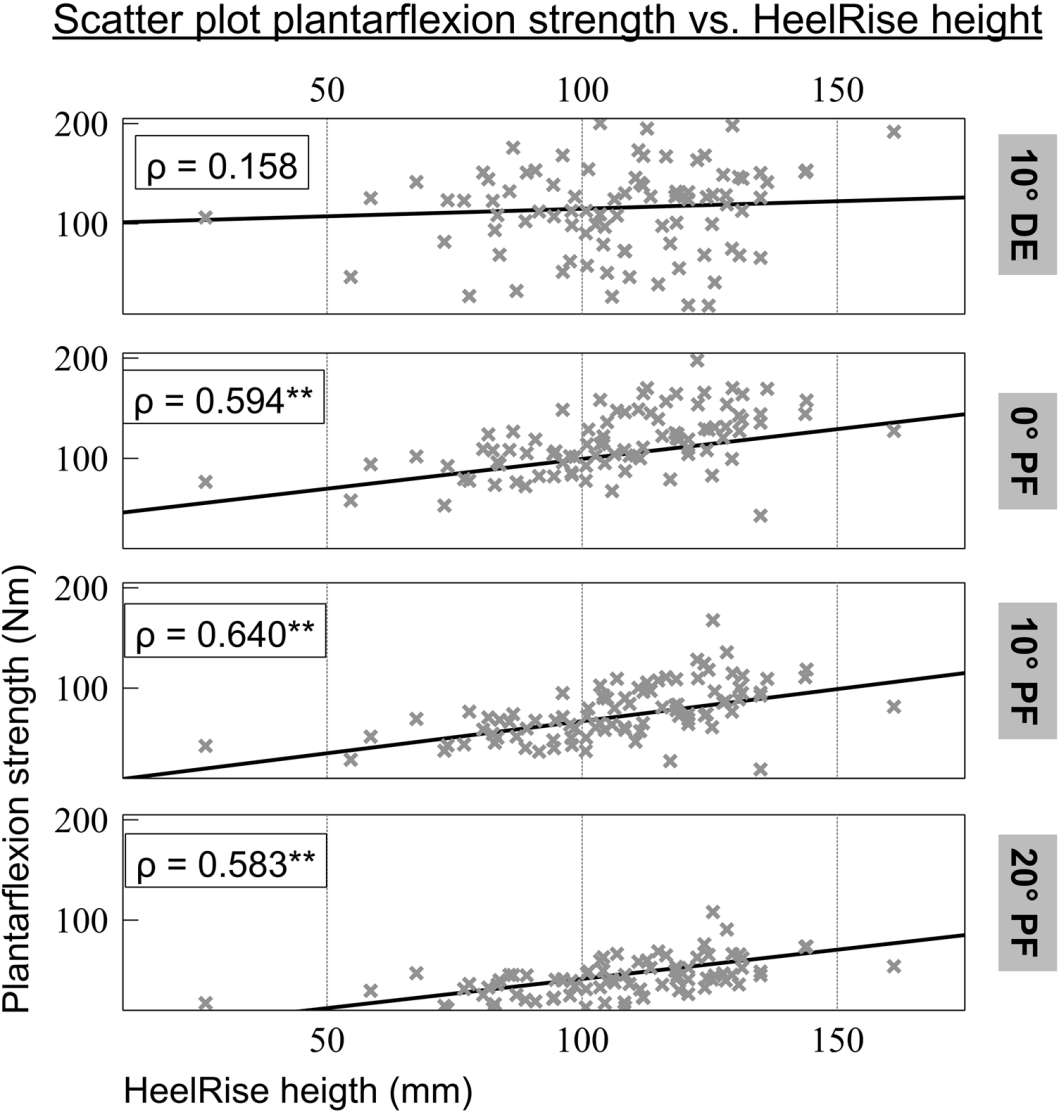
Correlation between plantarflexion strength and heel-rise height. *PF* plantarflexion, *DE* dorsal extension

Correlation analysis (Spearman’s rho) showed significant correlations between isokinetic plantarflexion torque across all plantarflexion angles and heel-rise height across all modalities (Table 6 and Fig. 6). These correlations were most pronounced during single-leg dynamic heel rise and plantarflexion torque at 10° of plantarflexion with Spearman’s rho at 0.64 (*p* < 0.01). To characterize the potential interaction of the investigated measures and the satisfaction during athletic performance, we calculated correlation analyses (Spearman’s rho) between the VAS of athletic performance, isokinetic strength, and heel-rise testing measures. The correlations were of weak or moderate strength, and they were comparable across the two subgroups, which is why they are displayed for the entire cohort. Noticeably, the correlations were constantly stronger between functional performance measures in heel-rise testing compared to isolated isokinetic strength testing. Table 6 shows the results of the correlation analyses.

**Table 6:** Correlation analysis of VAS athletic performance and functional performance measures

Parameter	Isokinetic plantarflexion strength				Heel-rise testing (HR_pos)			
	*F*Max @20°PF	*F*Max @10°PF	*F*Max @0°PF	*F*Max @10°DF	SLD	SLS	BD	BS
VAS athletic performance	**.24**	**.23**	**.23**	.13	**.27**	**.38**	**.27**	**.21**
Sign	**.02**	**.02**	**.02**	.2	**.009**	** < .001**	**.008**	**.037**

Bold indicates significant difference

*FMax*  maximum peak torque, *PF* plantarflexion, *DF* dorsiflexion, *HR_pos* heel-rise height above toe marker, *SLD* single-legged dynamic, *SLS* single-legged static, *BD* bipedal dynamic, *BS* bipedal static

## Discussion

In this study, we assessed midterm functional performance following surgical repair of the ruptured Achilles tendon. We implemented a progressive testing protocol to assess isokinetic strength deficits according to plantarflexion angle as previously suggested in the literature [25]. Furthermore, we examined whether potential deficits would reflect upon functional performance using a novel method of heel-rise testing as well as established gait analysis. We further compared these measures between two different techniques of surgical repair after acute AT rupture. Patient-reported outcome showed high satisfaction (VAS 8/10), and the side-to-side deficit in plantarflexion strength was found to be an average 10.2%, noticeably with an unequal distribution across the ROM during plantarflexion which was reflected into functional performance such as a lower turning point and a reduced maximum height during heel-rise testing.

### Patient-reported outcome

Generally, the PRO measures showed high satisfaction and good to very good scores on the ATRS. In detail, the results reveal that AR may lead to a slightly better subjective function than CR. However, it must be stated that the achieved results after AT rupture in an athletic population are overall satisfying at VAS for activities of daily living greater than 8/10 and an ATRS above 82 (CR) and 90 (AR). Moreover, subjective performance during sportive activities has also been found to be satisfactorily in both groups (VAS > 8/10). Correlation analyses further suggest that the significantly higher subjective function during sportive activities may be due to the more symmetric functional performance following AR with less side-to-side differences in MPTA. This difference may be crucial, e.g., for those athletes, relying on end-range plantarflexion strength during running, sprinting, or jumping [5, 25]. Overall, our findings are comparable with those previously reported in the literature where an average ATRS of 80–85 has been reported for surgical and conservatively treated patients [2, 3, 24, 31]. Interestingly, the accelerated rehabilitation seems to improve patient-reported outcome measures [24]. Since the VAS scores showed higher correlations with the heel-rise testing compared to isokinetic strength, it allows for the interpretation that functional performance is more important for the subjective outcome than the absolute strength.

### Isokinetic strength measurement

Recent studies manifested that soleus atrophy and subsequent loss of end-range plantarflexion torque seem to persist as a relevant problem after AT rupture regardless of the treatment method (surgical vs. non-surgical) or the exact surgical technique [5, 11, 25]. Strength deficits of 12–30% compared to the healthy leg have been described in the available literature [11, 12]. The differences in peak torque in this study were comparable to the values found in the literature [25, 29]. The significant differences in MPTA elicit that the distribution of strength is inhomogeneous. We concluded that the force–length relationship of the muscle–tendon unit remains altered and will not return to the previous muscle–tendon complex configuration which is of high relevance in daily locomotor activities [32, 33].

Side-to-side difference appeared to be more pronounced in the subanalysis with reference to the plantarflexion angle, where it was shown that with increasing end-range plantarflexion the deficits become greater (see Table 3 and Fig. 4). This underlines earlier findings that the exact interpretation of isokinetic strength testing in athletic performance testing should include at least maximum peak torque angle (MPTA), and it requires a detailed analysis of the distribution of strength across the ROM [11, 12, 25].

### Heel-rise test

In Heel-rise testing, this study showed that significant side-to-side differences persist regardless of the surgical technique. While there was no significant difference in the lower turning point (HR_neg) in any of the modalities, the highest height achieved (HR_pos) was significantly different in all of the scenarios. Interestingly, even in bipedal heel rise significant deficits on the operated legs were observed; thus, if it were only a deficit in strength, we would have expected a comparable performance of both legs in bipedal heel rise. Since there are significant differences in heel-rise height even though only half the weight is put on each leg, we must assume that structural changes regarding tendon elongation or alterations of the muscle–tendon unit with its strength–length relationship persist. In addition, the fact that there was a significantly greater deficit in heel-rise height (HR_pos) during single-leg performance underlines that the previously described deficit in end-range plantarflexion during isokinetic testing transfers into analogue deficits during functional performance.

Moreover, even though the lower turning point (HR_neg) did not reach significant differences all of the turning points were lower compared to the healthy side. In combination with the lower achievable height (HR_pos) and comparable values for the total distance, this suggests that the entire range of motion is shifted to more dorsiflexed position following AT rupture and repair. This is in line with the differences in maximum peak torque angle, and it further underlines the alteration of the triceps surae lever arm trajectories described following AT rupture [24, 34]. In our view, this adds a novel facet to the interpretation of a functional and structural elongation of the muscle–tendon unit in AT repair. [14, 25, 34]

As mentioned above, significant side-to-side differences were found in single-legged as well as in bipedal performance testing [13, 14]. When performing bipedal heel-rise testing a strength deficit could have been compensated for by the healthy side. Secondly, a neurological activation failure or inhibitory mechanisms as part of this specific performance deficit has been discussed, but additional data of this study including neuromuscular activation via electromyography registration show otherwise [34].

When comparing the two surgical techniques, LSIs in positive heel-rise height were significantly higher in AR compared to CR, underscoring the interpretation that AR leads to a more balanced recovery of functional performance. Potentially, this is due to the anatomical reconstruction of the twisted macrostructure of the AT and the elaborate integration of the ruptured soleus part leading to maximum pretensioning. The literature suggests that the role of the postoperative rehabilitation scheme is not as decisive more than one year post-surgery [9, 24]; however, an additional effect will require further evaluation.

As a final commentary, the novel setup for heel-rise testing used in this study proved to be viable and easy to apply. In our view, the data obtained using 3D motion analysis with a set of three markers offer a much more detailed analysis of the functional performance warranting the additional effort.

### Gait analysis

The finding that walking remains altered after AT repair has been previously described in the literature [35, 36]. During gait analysis in this study, we assessed parameters of subtle gait asymmetry like weight shift from rearfoot to forefoot, which previously served as good clinical indicators of subtle deficits in AT pathologies [35]. Despite the large size of the group, the observed differences were not significant, except the resting time of the hindfoot, which—as a biomechanical correlate—shows that the shift from rearfoot to forefoot occurs later during the gait cycle. Comparable measures have been described in the literature for patients recovering from non-surgical treatment after AT rupture as well as in Achilles tendinopathy [36, 37]. However, the detailed analysis of the neuromuscular activation did show significant differences in the EMG pattern, which is reported separately.

### Surgical technique

According to our initial hypothesis, end-range plantarflexion strength was improved in the anatomical reconstruction technique. The AR technique focuses on reintegration of the soleus muscle coupled with a pretensioning of the triceps surae complex while respecting the anatomical twist of the tendon. Potentially, this leads to a more symmetric, e.g., physiological, distribution of strength across the entire ROM. Contrarily, the finding that peak torque was produced at a more dorsiflexed angle in CR may allow for the interpretation that the muscle–tendon unit in the operated leg is effectively longer. On a functional level, this resulted in a more symmetric performance also during heel-rise testing and gait analysis in the AR group.

### Limitations 

One limitation of the study is the difference in group size, and another limitation is the number of tests. We therefore chose a conservative statistical approach and Bonferroni-adjusted alpha levels to avoid over-interpretation of the results. Furthermore, to the best of our knowledge, the ATRS has not been validated in German language, but it has been used in other studies of German-speaking populations before.

In our view, the difference in the rehabilitation protocols must be respected, but the most recent available literature yields that functional vs. conservative rehabilitation does not result in significant differences regarding tendon lengthening [24]. Furthermore, we would like to suggest that this analysis should be extended to a cohort of conservatively treated patients in order to critically evaluate the extent to which these findings may be generalized to AT rupture as a whole.

## Conclusions

The observed differences at 3.5 years following surgical repair of the AT underline the substantial chronic impairment following this injury, which restricts the force transmission and energy storage within the muscle–tendon complex and limits future sportive ability. Despite several deficits in strength and functional performance, the patients report a decent subjective function. The novel method of evaluating heel-rise testing will be of additional value in assessing functional performance deficits of the triceps surae unit. The additional evaluation of the neuromuscular activation pattern allows for a sound analysis of the persisting deficits. It yields potential fields of improvement like surgical focus on anatomical reconstruction of the tendon aiming to improve end-range plantarflexion strength.

## Supplementary Information

Below is the link to the electronic supplementary material.

Supplementary file1 (DOCX 20 KB)
