# Supplementary material for: Midterm functional performance following open surgical repair of acute Achilles tendon rupture
Source: Arch Orthop Trauma Surg. 2021 Jan 23;142(7):1337–49. doi: 10.1007/s00402-020-03746-3 (PMC9217896; doi:10.1007/s00402-020-03746-3)
Supplement: Supplementary file 1 — Supplementary file1 (DOCX 20 KB) [file 402_2020_3746_MOESM1_ESM.docx]

## - Supplementary Data -

### Table 1: Isokinetic strength analysis across the subgroups

| Parameter | AR  Mean ± SD | | CR  Mean ± SD | |
| --- | --- | --- | --- | --- |
|  | OP | NOP | OP | NOP |
| MPTA Plantarflexion (°) | -7.4±5.5 | -6.6±3.2 | -8.9±4.7 | -5.6±4.4 |
| FMax PF ROM (Nm) | 146.8±29.8 | 157.8 ±34.7 | 133.4±28.7 | 141.6±27.2 |
| FMax @ 20° PF (Nm) | 42.6±15.9 | 48.5 ±20.2 | 31.6±14.8 | 43.6±17.3 |
| FMax @ 10° PF (Nm) | 75.1±22.1 | 90.0±28.7 | 64.9±20.2 | 82.3±22.6 |
| FMax @ 0° PF (Nm) | 114.7±25.1 | 135.5 ±34.3 | 105.5±26.7 | 121.3±26.8 |
| FMax @ 10° DF (Nm) | 113.7±51.8 | 135.5±34.3 | 111.4±41.1 | 105.8±44.1 |
| MPTA Dorsiflexion (°) | 15.0±4.4 | 14.4±4.4 | 11.1±3.6 | 12.9±4.2 |
| FMax DF ROM (Nm) | 48.9±9.8 | 47.0±9.7 | 45.1±8.9 | 41.3±9.7 |

*AR=anatomical reconstruction, CR=conventional reconstruction, DF=dorsiflexion, FMax= maximum torque, MPTA=maximum peak torque angle (negative values = dorsiflexion), Nm=Newtonmeter, NOP= non-operated leg, OP= operated leg, PF=plantarflexion, ROM=Range-of-Motion, SD=standard deviation.*

| Parameter | | Total Cohort  Mean±SD (mm) | | AR  Mean±SD (mm) | | CR  Mean±SD in mm | |
| --- | --- | --- | --- | --- | --- | --- | --- |
|  |  | OP | NOP | OP | NOP | OP | NOP |
| Bipedal static | HR_tot | 179.6±21.9 | 185.8±19.4 | 179.3±23.7 | 184.3±20.3 | 180.2±20.7 | 186.2±18.9 |
|  | HR_pos | **120±16** | **126.5±14.3** | **123.9±13.0** | **128.3±13.4** | **112.4±18.1** | **123.2±15.4** |
|  | HR_neg | 60.2±17.9 | 58.0±15.3 | 57.5 ±17.3 | 56.1 ±18.0 | 65.3 ±17.5 | 61.3 ±12.4 |
| Bipedal dynamic | HR_tot | 186.6±17.9 | 193.0±15.4 | 188.2±18.7 | 192.8±17.0 | 184.9±17.6 | 191.1±13.4 |
|  | HR_pos | **122.8±14.4** | **130.9±15.2** | 126.0±14.1 | 134.6±15.4 | **116.9±14.1** | **125.7±12.6** |
|  | HR_neg | 64.2±17 | 62.6±18 | 62.1 ±17.2 | 60.1 ±17.3 | 70.8 ±15.7 | 67.4 ±16.9 |
| Single-leg static | HR_tot | **153.1±25.6** | **164.8±20.1** | 152.5±26.9 | 164.2±22.2 | **154.1±25.4** | **168.5±15.9** |
|  | HR_pos | **107.0±17.8** | **117.7±13.8** | **109.6±19.0** | **119.4±15.3** | **102.5±20.0** | **117.3±10.0** |
|  | HR_neg | 47.6±19.6 | 46.3±14.4 | 44.6 ±18.8 | 43.0 ±13.1 | 52.1 ±16.9 | 47.9 ±13.0 |
| Single-leg dynamic | HR_tot | **149.5±24.2** | **167.0±18.9** | 152.4±26.1 | 164.3±21.2 | **146.5±23.9** | **170.4±15.4** |
|  | HR_pos | **99.4±23.9** | **116.9±13.9** | **103.7±22.8** | **118.0±16.1** | **90.8 ±25.4** | **116.2±10.2** |
|  | HR_neg | 50.8±18.6 | 51.3±16.2 | 51.9 ±16.8 | 47.0 ±12.1 | 53.8 ±20.1 | 54.3 ±18.1 |
| AR= Anatomic reconstruction technique, CR=conventional technique, SD= standard deviation. OP = operated leg, NOP= contralateral healthy leg, HR_tot = total heel rise length, HR_pos= heel rise height above toe marker and HR_neg= heel rise height below toe marker.  **Bold values** = statistical significance, within-group using matched pair t-test, total group using single-factor ANOVA and Bonferroni-adjusted alpha level with p<0.017. | | | | | | | |

### Table 2 - Heel-Rise Testing results

Table 3 – Correlations between ground reaction force during walking and plantarflexion strength

| Parameter | Ground reaction force during push-off | |
| --- | --- | --- |
|  | **Barefoot** | **Shoes** |
| FMax total | **.27**** | **.24*** |
| FMax @20°’PF | .17 | **.30**** |
| FMax @10°PF | **.21*** | **.29**** |
| FMax=Maximum peak torque, PF=plantarflexion, DF=dorsiflexion. *=p<0.05, **p<0.01 | | |
